# Supplementary material for: Prognostic Value of Genetic Alterations in Elderly Patients with Acute Myeloid Leukemia: A Single Institution Experience
Source: Cancers (Basel). 2019 Apr 22;11(4):570. doi: 10.3390/cancers11040570 (PMC6520979; doi:10.3390/cancers11040570)
Supplement: Supplementary file 1 [file cancers-11-00570-s001.pdf]

Supplementary Materials

Prognostic Value of Genetic Alterations in Elderly Patients with Acute Myeloid Leukemia: A Single Institution Experience

Maël Heiblig, Hélène Labussière-Wallet, Franck Emmanuel Nicolini, Mauricette Michallet, Sandrine Hayette, Pierre Sujobert, Adriana Plesa, Marie Balsat, Etienne Paubelle, Fiorenza Barraco, Isabelle Tigaud, Sophie Ducastelle, Eric Wattel, Gilles Salles and Xavier Thomas

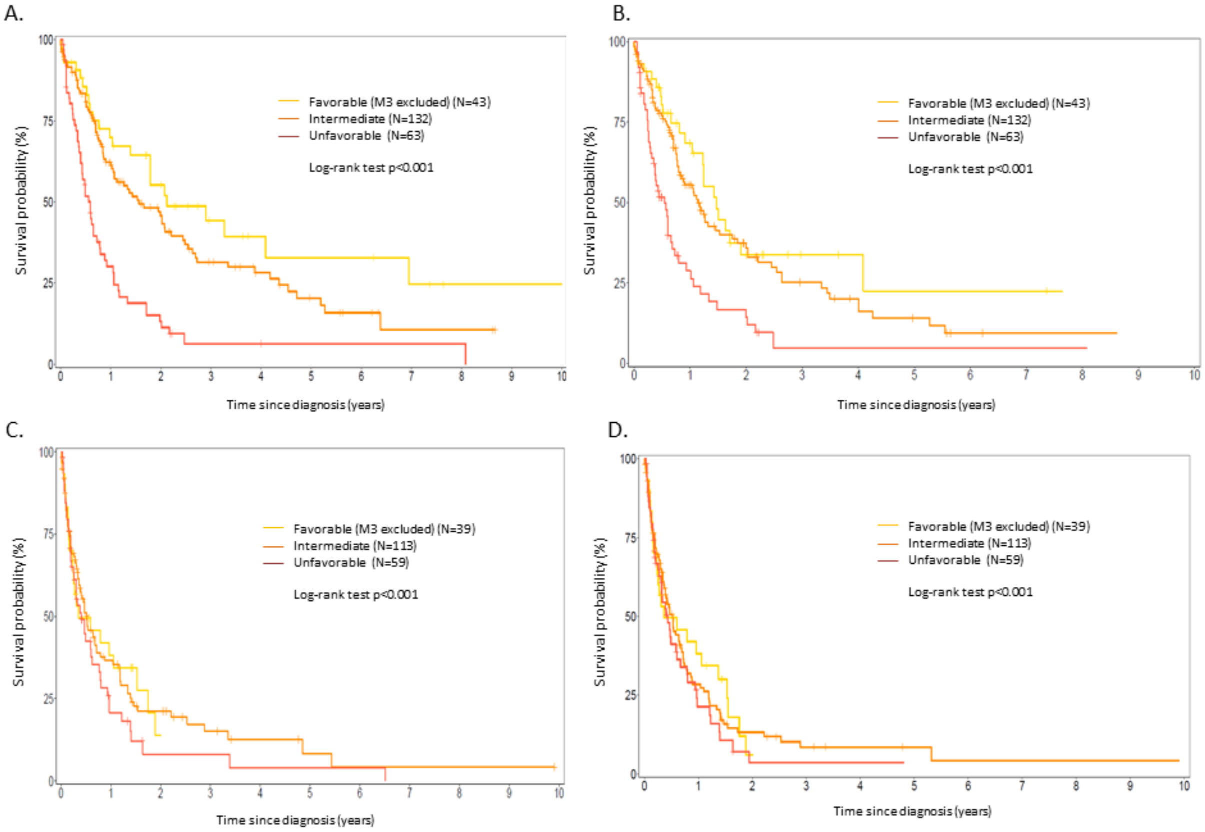

Figure S1. Survival curves according to age and ELN risk groups. (**A**) Group 1 overall survival according to the risk group; (**B**) Group 1 progression free survival according to the risk group; (**C**) Group 2 overall survival according to the risk group; (**D**) Group 2 progression free survival according to the risk group.

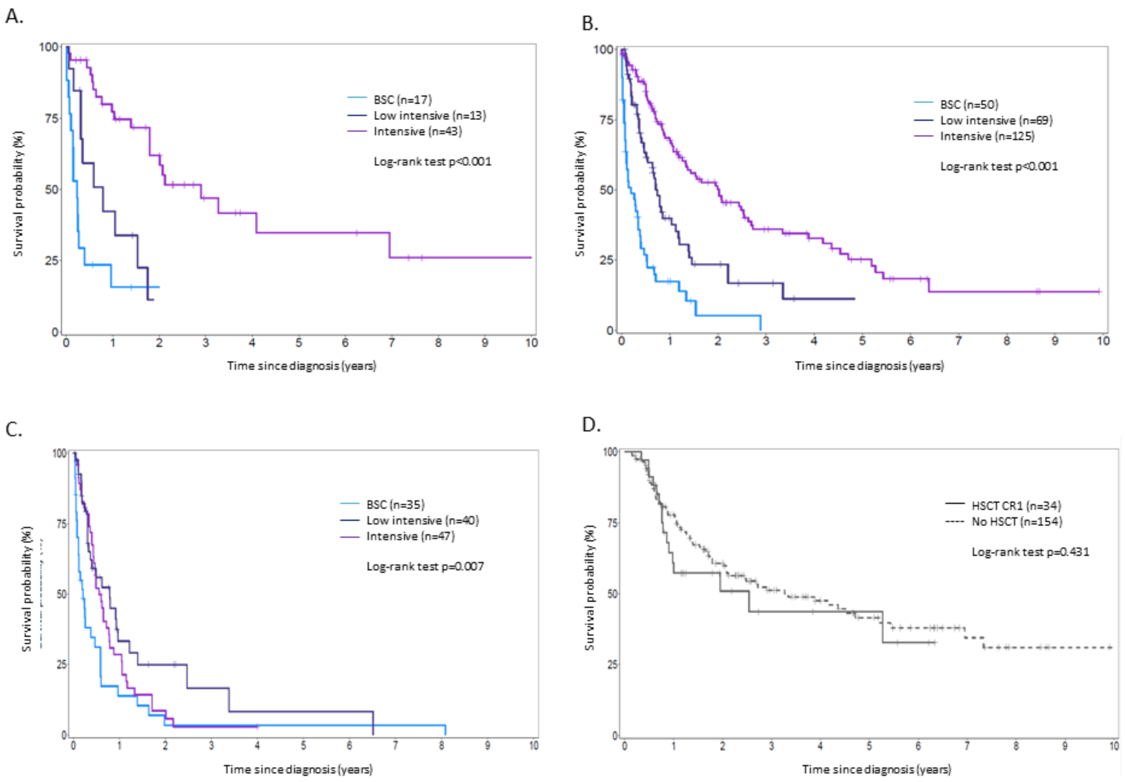

Figure S2. Survival curves according to treatment intensity and ELN risk groups**.** Overall survival of the entire cohort according to treatment intensity in (**A**) favorable, (**B**) intermediate and (**C**) unfavorable risk group. (**D**) Overall survival of patients allotransplanted in CR1 vs. those without HSCT.

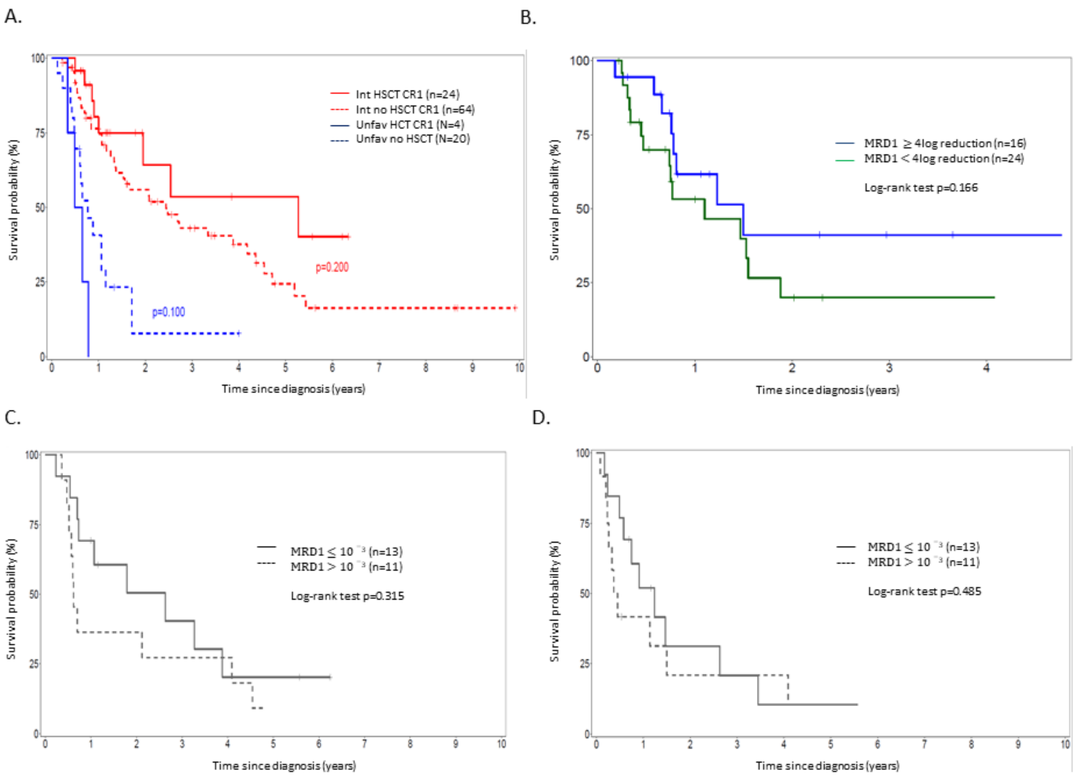

Figure S3. Survival curves according to *NPM1*/*WT1* molecular response and HSCT impact on survival. (**A**) Overall survival in intermediate and unfavorable risk group patients according to their HSCT status, (**B**) Progression free survival in *NPM1+* patients according to *NPM1* MRD1 log reduction, (**C**) Overall survival in *WT1* overexpressing patients according to *WT1* MRD1 absolute

value, (**D**) Progression free survival in *WT1* overexpressing patients according to *WT1* MRD1 absolute value.

Table S1. Cytogenetics and molecular demographics among ELN 2010 risk groups.

**Risk group**	**Karyotype**	**Total (*n* = 474)**	**Group1: 60–69 yo (*n* = 249)**	**Group2: 70+ yo (*n* = 225)**
Favorable risk group, *n* (%)	Normal karyotype with *NPM1* mutation without *FLT3-ITD* or *FLT3-ITD* ratio <0.10	62 (13.1%)	37 (14.8%)	25 (11.1%)
	CEBPα double mutated	2/145 (1.4%)	0/83	2/62 (3.2%)
	CBFβ and CBFα AML	10 (2.1%)	7 (2.8%)	3 (1.3%)
	t(15;17)	30 (6.3%)	16 (6.4%)	14 (6.2%)
Intermediate risk group, *n* (%)	NK *NPM1-/FLT3-*	102 (21.5%)	58 (23.3%)	44 (19.5%)
	NK *NPM1+/FLT3-ITD+*	29 (6.1%)	15 (6%)	14 (6.2%)
	NK *NPM1-/FLT3-ITD+*	15 (3.1%)	9 (3.6%)	6 (2.7%)
	t(3;5)(q21~25;q31~35)	0	0	0
	t(9;11)(p21~22;q23)	4 (1%)	2 (1%)	2 (1%)
	t(11;19)(q23;p13)	1 (<1%)	1 (<1%)	0 (<1%)
	t(6;9)(p23;q34) without *FLT3*-ITD	0	0	0
	Non monosomal complex karyotype	13 (2.7%)	7 (2.8%)	6 (2.7%)
	All other karyoypes	88 (18.6%)	40 (16.1%)	48 (21.3%)
Unfavorable risk group, *n* (%)	3q abnormalities [excluding t(3;5)(q21~25;q31~35)]	5 (1.1%)	3 (1.2%)	2 (<1%)
	inv(3)(q21q26.2) ou t(3;3)(q21;q26.2)	2 (<1%)	1 (<1%)	1 (<1%)
	t(6;11)(q27;q23)	2 (<1%)	1 (<1%)	1(<1%)
	t(10;11)(p11~13;q23)	1 (<1%)	0	1 (<1%)
	t(11q23) except t(9;11)(p21~22;q23) and t(11;19)(q23;p13)	6 (1.3%)	1 (<1%)	5 (2.2%)
	add(5q), del(5q), −5	2 (<1%)	0	2 (<1%)
	−7, add(7q)/del(7q)	8 (1.7%)	4 (1.6%)	4 (1.8%)
	−17/17p abnormality	15 (3.2%)	6 (2.4%)	9 (4%)
	Monosomal karyotype	79 (16.7%)	30 (12.4%)	49 (21.8%)
	t(6;9)(p23;q34) with FLT-ITD	1 (<1%)	1 (<1%)	0

Table S2. Overall outcome according to the risk group independently of treatment intensity (excluding APL in favorable group).

Age/risk group	Median OS, Months (95% CI)	1 year OS Probability, % (95% CI)	*p*-Value	Median PFS, Months (95% CI)	1 year PFS Probability % (95% CI)	*p*-Value
Overall	10.6 (8.7–12.8)	47.3 (42.8–52.2)	-	9.3 (8.1–11.1)	43.6 (39.1–48.6)	-
Group 1	15.9 (12.2–23.8)	56.4 (50.4–63)	<0.001	12.7 (9.8–15.9)	52.7 (46.6–59.6)	<0.001
Group 2	6.31 (4.8–9.4)	36.3% (30.2–43.7)		6.4 (4.8–8.5)	32.8 (26.8–40.2)	
**Risk group**						
Favorable	21.1 (9.4–25.4)	56.8 (46.1–69.9)		14.8 (9.6–18.5)	55.9 (45–69.3)	
Intermediate	10.8 (8.5–15.1)	49.2 (42.9–56.4)	<0.001	9.2 (8.2–12.5)	42.8 (36.5–50.1)	<0.001
Unfavorable	5.9 (4.8–7.5)	25.8 (18.5–35.8)		5.2 (4.4–7.1)	25.1 (17.7–35.6)	
**Survival according to risk group in age group 1**						
Favorable	25.4 (21.4–NR)	69.9 (56.9–85.8)		17.6 (14.7–NR)	68.5 (55–85.3)	
Intermediate	18.3 (12.6–29.3)	59.9 (51.6–69.6)	<0.001	13.7 (9.8–19.1)	54.9 (46.3–65)	<0.001
Unfavorable	6.8 (5.1–9.5)	30.1 (20.2–44.7)		6.5 (4.4–8.6)	28.7 (18.6–44.3)	
**Survival according to risk group in age group 2**						
Favorable	4.2 (2.9–21.1)	38.1 (23.8–61)		4 (2.9–18.3)	37.5 (22–59.7)	
Intermediate	6.3 (4.6–8.5)	36.5 (28–47.5)	0.55	6.1 (4.6–8.2)	28.4 (20.6–39.2)	0.69
Unfavorable	4.8 (3.1–9.2)	20.7 (11.6–36.9)		5.1 (3.7–9.4)	21.3 (12–37.9)	

Legend: NR = not reached, OS = overall survival, PFS = progression free survival, yo = years old.

Table S3. Clinical outcome according to the risk group and the initial treatment type after removing APL patients.

Risk group	Treatment Intensity	Total				Group1: 60–69 yo (*n* = 260)				Group2: 70+ yo (*n* = 235)			
		Median OS, Months (95% CI)	*p*-Value	Median PFS, Months (95% CI)	*p*-Value	Median OS, Months (95% CI)	*p*-Value	Median PFS, Months (95% CI)	*p*-Value	Median OS, Months (95% CI)	*p*-Value	Median PFS, Months (95% CI)	*p*-Value
	Intensive	32.8 (22–NA)		16.5 (14.8–NR)		34.8 (22–NR)		18 (14.8–NR)		24.1 (1–NR)		12.5 (1.1–NR)	
**Favorable**	Low intensive	12.6 (4–NR)	<0.001	8.9 (3.7–NR)	<0.001	- *	<0.001	- *	NE	12.6 (4–NR)	0.16	8.9 (3.7–NR)	0.1
	BSC	2.7 (2–NR)		2.7 (1.6–16.3)		- *		- *		2.7 (2–NR)		2.7 (1.6–NR)	
	Intensive	23.8 (16–32.3)		14.2 (10.6-21.1)		24.2 (16.5–32.6)		15 (12.5–23.85)		16.1 (10.6-NR)		10.5 (6.9–NR)	
**Intermediate**	Low intensive	8.5 (6.1–14.2)	<0.001	8.15 (5.6–10.3)	<0.001	9.8 (8.4–NR)	<0.001	8.8 (8.9–NR)	<0.001	7.7 (5.5–16.7)	<0.001	7.7 (5–10.3)	0.02
	BSC	2.2 (1.15–4.6)		2.2 (1.15–4.6)		3.4 (1.5–5.6)		3.4 (1.5–5.6)		0.7 (0.3–NR)		0.7 (0.3–NR)	
	Intensive	6.8 (5–10)		6.5 (4.4–8.6)		6.8 (5–10)		6.4 (4.4–9.5)		7.4 (2–NA)		6.8 (1.8–NA)	
**Unfavorable**	Low intensive	9.8 (4–17)	0.004	8.4 (4.5–16.8)	0.007	11.1 (4–NR)	0.15	10.2 (4.5–NR)	0.1	9.0 (4–17)	0.009	7 (3.9–16.8)	0.02
	BSC	2.9 (1.4–7.1)		2.5 (1–7)		3 (1–NR)		2.9 (1.3–NR)		2.5 (1–7)		2.3 (1–7)	

* No patients received low intensive or BSC. Legend: BSC = best supportive care, NE = non evaluable, NR = not reached, OS = overall survival, PFS = progression free survival, yo = years old.

Table S4. Clinical characteristics and outcome of *NPM1+* and *WT1 OE* patients according to their MRD1 response after standard induction.

***NPM1* Mutated Patients**	**MRD1 ≥ 4 log (N = 16)**	**MRD1 < 4 log (N = 24)**	***p*-Value**
Median age, years (range)	64.1 (62.3–68.2)	64.9 (60–70.1)	0.87
PS > 2, *n* (%)	1/13 (7.7%)	4/23 (17.4%)	0.4
LDH (range)	427 (263–821)	828 (205–5800)	0.09
NK, *n* (%)	15/16 (93.7%)	21/24 (87.5%)	0.24
FLT3-ITD+, *n* (%)	3/16 (18.7%)	14/24 (58.3%)	0.01
Median OS, months (range)	31.6 (8.8–NR)	7.4 (6.4–NR)	0.001
***WT1* Overexpressing Patients**	**MRD1 ≤ 10^{-3} (N = 13)**	**MRD1 > 10^{-3} (N = 11)**	***p*-Value**
Median age, years (range)	66 (60–69.7)	66.5 (60–71.2)	0.91
PS > 2, *n* (%)	1/13 (7.7%)	1/11 (9.1%)	0.75
LDH (range)	852 (346–1660)	1135 (202–1800)	0.78
NK, *n* (%)	9/13 (69.2%)	9/11 (81.8%)	0.47
FLT3-ITD+, *n* (%)	3/13 (23.1%)	5/11 (45.4%)	0.45
Median OS, months (range)	25.4 (21.52–NR)	12.85 (8.4–24.15)	0.325

Legend: LDH = lactacte deshydrogenase, MRD1 = post induction minimal residual disease by RT-qPCR, NK = normal karyotype, NR = not reached, OE = overexpression, OS = overall survival, PS = performance status.

Table S5. Univariate analysis.

**Variable**	**Modality**	**OS**			**PFS**		
		**HR**	**95% CI**	**Cox *p*-Value**	**HR**	**95% CI**	**Cox *p*-Value**
Age at diagnosis (yo) *		1.02	(0.98–1.06)	0.317	1.01	(0.97–1.05)	0.624
Age groups	Group1: 60–69 yo	1.00	-	-	1.00	-	-
	Group2: 70+ yo	0.83	(0.51–1.34)	0.437	0.89	(0.57–1.40)	0.613
Gender	M	1.00	-	-	1.00	-	-
	F	1.05	(0.76–1.46)	0.767	1.04	(0.75–1.44)	0.829
PS	0	1.00	-	-	1.00	-	-
	1	0.92	(0.64–1.32)	0.660	1.05	(0.74–1.48)	0.806
	2 or more	1.85	(1.18–2.90)	0.007	1.38	(0.86–2.21)	0.176
WBC *		1.01	(1.00–1.01)	<0.001	1.01	(1.00–1.01)	0.001
Hemoglobin *		1.00	(0.99–1.00)	0.684	1.00	(0.99–1.01)	0.983
Platelets *		1.00	(1.00–1.00)	0.297	1.00	(1.00–1.00)	0.405
Polymorphonuclear cells		1.01	(1.00–1.01)	0.169	1.01	(1.00–1.02)	0.035
Peripheral blasts (%)		1.01	(1.00–1.01)	0.002	1.01	(1.00–1.01)	0.002
Medullar blasts (%)		1.00	(0.99–1.01)	0.837	1.00	(0.99–1.01)	0.807
LDH *		1.00	(1.00–1.00)	0.504	1.00	(1.00–1.00)	0.131
AML type	De novo	1.00	-	-	1.00	-	-
	Secondary	1.54	(1.12–2.12)	0.008	1.68	(1.23–2.29)	0.001
	No	1.00	-	-	1.00	-	-
Extramedullar localization	No	1.00	-	-	1.00	-	-
	SNC	1.19	(0.58–2.43)	0.635	2.03	(1.28–3.20)	0.002
Normal karyotype	Yes	1.00	-	-	1.00	-	-
	No	1.13	(0.81–1.57)	0.476	0.87	(0.62–1.21)	0.396
	Unknwown	1.79	(0.92–3.48)	0.084	1.60	(0.77–3.34)	0.208
ELN 2010 risk group	Favourable	1.00	-	-	1.00	-	-
	Intermediate	2.40	(1.52–3.81)	<0.001	3.07	(1.91–4.94)	<0.001
	Unfavourable	7.89	(4.61–13.51)	<0.001	8.39	(4.92-14.32)	<0.001
	Unknown	3.98	(1.70–9.34)	0.001	5.45	(2.28–12.99)	<0.001
Response	CR	1.00	-	-	1.00	-	-
	Refractory	3.53	(2.51–4.95)	<0.001	2.35	(1.69–3.26)	<0.001
	Not evaluated	12.64	(4.74–33.72)	<0.001	9.41	(3.57–24.81)	<0.001
NPM1	Not mutated	1.00	-	-	1.00	-	-
	Mutated	0.83	(0.58–1.20)	0.328	0.81	(0.56–1.17)	0.263
FLT3-ITD	Not mutated	1.00	-	-	1.00	-	-
	Mutated	1.92	(1.29–2.88)	0.001	1.79	(1.14–2.82)	0.012
FLT3-TKD	Not mutated	1.00	-	-	1.00	-	-
	Mutated	1.39	(0.64–3.01)	0.402	1.54	(0.70–3.38)	0.282
WT1	Normal	1.00	-	-	1.00	-	-

	Overexpressed	1.16	(0.82–1.63)	0.403	1.08	(0.76–1.51)	0.674
MECOM1	Normal	1.00	-	-	1.00	-	-
	Overexpressed	1.12	(0.74–1.70)	0.586	1.11	(0.73–1.68)	0.623
IDH	Not mutated	1.00	-	-	1.00	-	-
	IDH1	0.62	(0.36–1.10)	0.103	0.81	(0.45–1.45)	0.479
	IDH2	0.24	(0.08–0.72)	0.011	0.20	(0.07–0.60)	0.004
DNMT3A	Not mutated	1.00	-	-	1.00	-	-
	Mutated	0.69	(0.38–1.24)	0.215	0.93	(0.52–1.66)	0.803
MLL-DUP	Not mutated	1.00	-	-	1.00	-	-
	Mutated	1.16	(0.62–2.17)	0.639	1.08	(0.56–2.11)	0.813
FLT3-ITD-NPM1+	Not mutated	1.00	-	-	1.00	-	-
	Mutated	0.30	(0.17–0.54)	<0.001	0.38	(0.22–0.68)	0.001
FLT3-TKD+NPM1+	Not mutated	1.00	-	-	1.00	-	-
	Mutated	0.25	(0.063–1.02)	0.053	0.55	(0.18–1.67)	0.292
MRD1 *NPM1* ≥ *4 log reduction*	Yes	1.00	-	-	1.00	-	-
	No	3.32	(1.42–7.77)	0.006	2.44	(1.03–5.73)	0.042
Genetic characteristics at treatement decision	Absent	1.00	-	-	1.00	-	-
	Present	0.25	(0.17–0.37)	<0.001	0.32	(0.21–0.49)	<0.001
HSCT in CR1	No	1.00	-	-	1.00	-	-
	Yes	1.25	(0.76–2.05)	0.376	4.26	(2.02–8.97)	<0.001

* Continuous variables.

Table S6. Interaction score of variables influencing survival.

Interaction	PFS		OS	
	Chisq	*p*-Value	Chisq	*p*-Value
Age group x treatment intensity	0.56	0.76	0.82	0.66
PS score x treatment intensity	5.98	0.55	5.56	0.59
ELN risk group classification x treatment intensity	10.53	0.10	8.92	0.18
FLT3-ITD status x treatment intensity	1.21	0.55	1.44	0.48
Genetic characteristics at treatment decision x treatment intensity	6.37	0.041	7.21	0.027

Legend: OS = overall survival, PFS = progression free survival, PS = performance status.

© 2019 by the authors. Licensee MDPI, Basel, Switzerland. This article is an open access article distributed under the terms and conditions of the Creative Commons Attribution (CC BY) license (http://creativecommons.org/licenses/by/4.0/).
